# Differences in STI/HIV Burden and Sexual Health Care–Seeking Behavior Among First- and Second-Generation Migrant and Western-Born Male Sex Workers Who Have Sex With Men in the Netherlands: A Retrospective Cohort Study

**DOI:** 10.1097/OLQ.0000000000001902

**Published:** 2023-11-13

**Authors:** Charlotte M.M. Peters, Ymke J. Evers, Carolina J.G. Kampman, Marie-José Theunissen-Lamers, Mark A.M. Van Den Elshout, Nicole H.T.M. Dukers-Muijrers, Christian J.P.A. Hoebe

**Affiliations:** From the ∗ Department of Social Medicine, Care and Public Health Research Institute (CAPHRI), Maastricht University/Maastricht UMC+, Maastricht; † Department of Sexual Health, Infectious Diseases and Environmental Health, Living Lab Public Health, South Limburg Public Health Service, Heerlen; ‡ Public Health Service Twente, Enschede; § Department of Sexual Health, Public Health Service GGD Brabant Zuidoost, Helmond; ¶ Department of Infectious Diseases, Public Health Service of Amsterdam, Amsterdam; ∥ Department of Sexual Health, Public Health Service of the Utrecht Region, Utrecht; ∗∗ Department of Health Promotion, Care and Public Health Research Institute (CAPHRI), Maastricht University/Maastricht UMC+; †† Department of Medical Microbiology, Infectious Diseases and Infection Prevention, Care and Public Health Research Institute (CAPHRI), Maastricht University Medical Centre (MUMC+), Maastricht, the Netherlands

## Abstract

**Background:**

Globally, migrant sex workers have a higher burden of sexually transmitted infections (STI)/human immunodeficiency virus (HIV). This large study aimed to assess demographics, STI/HIV burden, and sexual health care–seeking behavior of first-generation migrant and second-generation migrant male sex workers who have sex with men (MSW-MSM) versus Western-born MSW-MSM.

**Methods:**

Coded STI clinic consultations (n = 6970) from 3116 individual MSW-MSM attending any Dutch STI clinic between 2016 and 2021 were included. First-generation migrant: born outside of northern/central/southern/western Europe/North America/Oceania. Second-generation migrant: ≥1parent born outside of northern/central/southern/western Europe/North America/Oceania. Multivariable logistic regression analysis assessed associations between MSW-MSM groups and STI in first consultation in the data. A Cox proportional hazard regression compared the incidence of a first repeat consultation between migration groups, stratified by STI in first consultation. All analyses were adjusted for age and urbanity of STI clinic region.

**Results:**

First-generation migrant MSW-MSM (n = 1085) were mostly born in Latin America (50%), whereas second-generation migrant MSW-MSM (n = 368) mostly originated from North Africa (30.4%). The proportion of STI diagnoses differed (33.2%, 29.3%, 23.3%; *P* < 0.001) between the first-generation migrant, second-generation migrant, and Western-born MSW-MSM. First-generation migrant MSW-MSM versus Western-born had an adjusted odds ratio of 1.6 (95% confidence interval, 1.3–1.9) of STI diagnosis in the first consultation. First-generation migrant MSW-MSM versus Western-born had an adjusted hazard ratio of 1.5 (95% confidence interval, 1.3–1.8) of having a first repeat consultation at any time, when stratified for no STI in the first consultation.

**Conclusions:**

The STI/HIV burden is high among all 3 MSW-MSM groups. First-generation migrants have higher odds of STI, but retention in care seems similar. Results highlight the importance of low-threshold STI testing and care for (migrant) MSW-MSM.

## Abstract 2

First-generation migrant male sex workers who have sex with men were found to be more likely get a sexually transmitted infection diagnosis and a repeat consultation than Western-born male sex workers who have sex with men in the Netherlands.

Western Europe has the largest population of migrant male sex workers in Europe, with migrants accounting for 46% of the male sex workers population.^1^ Male sex workers are defined as men who have sex with men (MSM) in exchange for money or goods. The migrant sex work population in the Western European region is highly diverse and mobile, with an average of 33 nationalities per Western European country and 82% of migrant sex workers reporting to have worked in multiple countries.^1,2^

Population mobility is an important determinant in the global epidemiology of sexually transmitted infections (STI), including human immunodeficiency virus (HIV).^3^ Because of their elevated likelihood of STI/HIV, it is deemed important that migrant male sex workers who have sex with men (MSW-MSM) have low-threshold access to sexual health care. However, European studies suggest that migrant MSW-MSM lack access to (sexual) health care services because their sociolegal position.^2,4^

Male sex workers who have sex with men are more likely to acquire STI/HIV than female sex workers (FSWs) and MSM.^5^ The process of migration, that is, going from a traditional home society to a more liberal society, has shown to increase sexual activity and increase chances to acquire STI/HIV of Central and East European MSM migrants.^6^ Furthermore, studies show that migrant MSM portray behavior, which makes them more vulnerable to HIV within the immediate postmigration period, and thus have an increased chance of acquiring STI/HIV after migration.^7,8^ Nonetheless, research on STI/HIV burden and sexual health care use of migrant MSW-MSM in Western Europe is scarce.^9^ It is unclear to what extent migrant MSW-MSM experience more adverse sexual health outcomes compared with nonmigrant MSW-MSM in Western Europe. Limited previous studies have not made a differentiation in migration generation (first- or second-generation migrant) and whether MSW-MSM's STI/HIV burden differs by migration generation. Furthermore, access to sexual health care services by migrant MSW-MSM in Western Europe has yet to be studied.

This current study used a large dataset from a national surveillance registry including patients of all STI clinics during a 6-year period in the Netherlands (2016–2021). We evaluated the representation of migrant MSW-MSM in all sexual health care consultations, their sociodemographic characteristics, chance and burden of STI/HIV, and sexual health care–seeking behavior among first- and second-generation migrant and Western-born MSW-MSM. Findings will inform sexual health care policy and guidelines for the male migrant sex worker populations and will provide understanding of the reach of this key population by the Dutch STI clinics.

## MATERIALS AND METHODS

### Study Design

In this retrospective cohort study, coded surveillance consultations of MSW-MSM were included from all outpatient Public Health STI clinics in the Netherlands (25 Public Health Services with 38 STI clinic locations), which were submitted between January 1, 2016, and December 31, 2021, via an electronic patient registry using a consultation code, to the National Institute of Public Health and the Environment. The publicly funded STI clinics are required to submit their consultations to the national institute; clients are allowed to opt out.

Consultation-level and individual-level data were extracted on sociodemographic characteristics, sexual behavior, and STI diagnosis. Sociodemographic characteristics and sexual behavior were obtained from a medical and sexual history patient questionnaire conducted by STI clinic nurses and registered in the electronic patient registry.

The STI clinics provide free of charge, anonymous, and confidential sexual health care to high-incidence STI groups including, among others, sex workers, individuals from STI-endemic countries, and MSM. According to the Dutch national STI-testing policy, MSM sex workers are advised to get tested for STI at least 4 times a year.^10^

All MSM are routinely tested for urogenital, anorectal, and oropharyngeal *Chlamydia trachomatis* and *Neisseria gonorrhoeae*, HIV, hepatitis B, and syphilis. Urine, self-collected anorectal swabs, and self- or provider-collected oropharyngeal swabs were tested for *C. trachomatis* and *N. gonorrhoeae*. Blood samples were tested for HIV, hepatitis B, and syphilis. The specimens were processed at different Dutch medical microbiological laboratories using commercially available diagnostic tests. After the first hepatitis B vaccination, testing for hepatitis B is omitted.

Of all consultations from the years 2016 to 2021 (n = 799,400), only consultations from sex workers registered as MSM were included (6970 consultations) in this study.

### Study Context

In 2022, 25.9% of the total of 17.7 million Dutch inhabitants had a migration background, of which 14% was not born in the Netherlands.^11^ The main countries of origin from Dutch inhabitants with a migration background were Turkey, Morocco, Suriname, Indonesia, Germany, and Poland.^11^ Suriname, Indonesia, and the Netherlands (NL) Antilles are former Dutch colonies, and Turkey and Morocco are former guest workers' countries.^12^ The islands that were part of the Netherlands Antilles are now still part of the Dutch Kingdom as separate countries or as special municipalities.

### Definitions

Male sex workers who have sex with men are defined as MSM in exchange for money or goods. Because the MSW-MSM included in this study are registered as male in the electronic patient registry, we assume that they are cisgender males; however, we are mindful that possibly not everyone identifies as a cisgender male.

Western-born is defined as born in Northern, Central, Southern and Western Europe, Oceania, or North America.

Although we are aware that the terms *immigrant* and *migrant* are both used in the literature and have a partly overlapping meaning, we choose to use the broader term migrant. First-generation migrant is defined as born in Eastern Europe, Latin America, Asia, Africa, Netherlands Antilles, or Suriname. Second-generation migrant is defined as Western-born with at least one parent who is born in Eastern Europe, Latin America, Asia, Africa, Netherlands Antilles, or Suriname. Level of education was categorized into the following: low, elementary and prevocational secondary; medium, senior general secondary, preuniversity, and secondary vocational; high, higher professional and university based on the definitions used by Statistics Netherlands.^13^

Urbanity was assigned to the STI clinic region based on regional data from Statistics Netherlands.^13^ If an STI clinic region had ≥900 inhabitants per kilometer squared, the region was assigned “high urban,” and if the region had <900 inhabitants per kilometer squared, the region was assigned “moderate to low urban.”

The variable condom use during anogenital sex has a “not applicable” option, because some MSW-MSM do not have anogenital sex. For alcohol/drug use during sex <6 months, the options “no” and “unknown” were merged together into “no.”

Burden of STI/HIV was measured with STI diagnosis, which was defined as being diagnosed with *C. trachomatis*, *N. gonorrhoeae*, infectious syphilis (primary syphilis, secondary syphilis, and early latent syphilis), infectious hepatitis B, or a newly diagnosed HIV infection. The first STI test consultation of a patient that appears in our data is considered as the first consultation. A repeat consultation was defined as the first STI test consultation >1 month after the first STI test consultation carried out by the same STI clinic in the available data. We excluded all consultations within 1 month after the previous consultation, to ensure that the consultation is not merely a follow-up to the previous consultation. Sexual health care–seeking behavior was measured by assessing repeat consultation(s) on an individual level.

Being lost to care is referred to when a patient is lost to follow-up in sexual health care after having a first consultation at any Dutch STI clinic.

### Data Analyses

Descriptive analyses were performed to describe proportions of sociodemographic characteristics, sexual behavior, and STI diagnosis in the first consultation, among continents and for one-time and repeat testers among first- and second-generation migrant and Western-born MSW-MSM. Sociodemographic characteristics and STI diagnoses were compared between first- and second-generation migrant and Western-born MSW-MSM using *χ*^2^ tests. If more than >2.5% was missing, a category “unknown” was created and reported in the results.

A logistic regression was performed to test associations between migration status and any STI diagnosis in the first consultation while adjusting for age and urbanity of the STI clinic region. As a sensitivity analysis, a similar analysis was performed with the outcome diagnosis of any STI excluding HIV in the first consultation. A Kaplan-Meier survival plot was performed to compare the proportion of repeat consultations among the 3 MSW-MSM groups, with a log-rank test comparing the 3 survival curves. Exposure time was defined as the time between the first consultation and the first repeat consultation or the end of the study period. A Cox proportional hazard regression was performed to assess the associations between the repeat consultations among the 3 MSW-MSM groups, adjusting for age, urbanity, and STI in the first consultation. Other registry variables were not adjusted for because of insufficient quality of the data, for example, too many missing values. The assessed hazard ratio (HR) shows the probability of an event happening in one group relative to the probability of an event happening in a control group over a unit of time.

An interaction term was added for MSW-MSM group and STI in the first consultation, stratifying the Kaplan-Meier and Cox proportional hazard analyses for STI in the first consultation if significant.

An *α* ≤0.05 was considered significant in all analyses. Statistical analyses were done using IBM SPSS Statistics (Version 27.0; Armonk, NY).

### Ethical Considerations

The Medical Ethics Committee of Maastricht University waived the requirement for ethical approval and written informed consent because the data used were coded, originated from standard care, and were analyzed anonymously (METC 2017-0251).

## RESULTS

This study included 6970 STI clinic consultations of 3116 unique MSW-MSM, of which 1085 were first-generation migrants, 398 second-generation migrants, and 1663 Western-born MSW-MSM. Sociodemographic characteristics of the study population are presented in Table 1. The median age was lower among second-generation MSW-MSM compared with first-generation migrant and Western-born MSW-MSM (*P* < 0.001). More first-generation migrant MSW-MSM visited STI clinics in a high urban region compared with second-generation migrant and Western-born MSW-MSM (*P* < 0.001). The level of education was largely unknown 1 (49.4%) for the first-generation migrants and similar for the second-generation migrant and Western-born MSW-MSM. Most first-generation migrant MSW-MSM were born in Latin America (50%), followed by Asia (15.3%) and Eastern Europe (14.9%), whereas the birth region of the second-generation migrant MSW-MSM their parent(s) was mostly North Africa (30.4%), followed by Suriname/Netherlands Antilles (28.8%) and Asia (21.5%).

**TABLE 1:** Sociodemographic Characteristics of the Study Population of (Migrant) Male Sex Workers Who Have Sex With Men (MSW-MSM) Who Visited an STI Clinic From 2016 to 2021 in the Netherlands (n = 3116)

Sociodemographic Characteristics Reported in First Consultation	First-Generation Migrant MSW-MSM (N = 1085)		Second-Generation Migrant MSW-MSM (N = 368)		Western-Born MSW-MSM (N = 1663)		*χ*^2^ Test, *P* Value
	% of Total	n	% of Total	n	% of Total	n	
Age, median (IQR), y	31 (26–39)		27 (23–34)		31 (25–44)		<0.001*
Level of education							n/a
High	21.1	229	41.0	151	41.8	695	
Middle	10.0	108	20.9	77	18.2	302	
Low	19.5	212	29.9	110	30.6	509	
Unknown	49.4	536	8.2	30	9.4	157	
Urbanity of STI clinic region^†^							**<0.001**
High urban	90.1	978	82.1	302	68.1	1132	
Moderate to low urban	8.8	96	17.4	64	30.1	500	
Birth region MSW-MSM/MSW–MSM's parent(s)					n/a		n/a
Latin America	50.0	543	8.7	32			
Asia	15.3	166	21.5	79			
Eastern Europe	14.9	162	5.2	19			
Suriname/Netherlands Antilles	9.7	105	28.8	106			
North Africa	5.9	64	30.4	112			
Sub-Sahara Africa	4.1	45	9.8	36			

Statistically significant *P* values in bold (* P* < 0.05).

*Analysis of variance test to compare means.

^†^Missing: 1.0% (11), 0.5% (2), and 1.9% (31), respectively.

Sexual behavior reported in the first consultation and diagnosis of STI in the first consultation of the study population are presented in Table 2.

**TABLE 2:** Sexual Behavior and Sexually Transmitted Infection (STI) Diagnosis in First Consultation of (Migrant) Male Sex Workers Who Have Sex With Men (MSW-MSM; n = 3116) Who Visited an STI Clinic From 2016 to 2021 in the Netherlands

	First-Generation Migrant MSW-MSM (N = 1085)		Second-Generation Migrant MSW-MSM (N = 368)		Western-Born MSW-MSM (N = 1663)		*χ*^2^ Test, *P* Value*
	% of Total	n	% of Total	n	% of Total	n	
**Sexual behavior reported in first consultation**							
No. sex partners <6 mo, median (IQR)	40.0 (10–152)		12.0 (6.0–30.0)		15.0 (6.0–30)		0.269^†^
Condom use during anogenital sex^‡^							**<0.001**
N/a	1.2	9	1.7	4	2.8	28	
Never	15.4	116	14.5	35	17.1	173	
Not always	48.7	368	61.0	147	58.0	585	
Always	34.7	262	22.8	55	22.1	223	
PrEP use^§,¶^							0.223
No	69.7	517	75.3	162	72.2	614	
Yes	30.3	225	24.7	53	27.8	236	
Sex with^∥^							**<0.001**
Men	74.5	808	57.9	213	56.2	935	
Men and women	23.2	252	40.2	148	42.8	712	
Alcohol/drug use during sex <6 mo							**0.006**
No	56.6	614	50.3	185	49.4	821	
Yes	43.4	471	49.7	183	50.6	842	
**STI diagnosis in first consultation**							
Any STI							**<0.001**
Yes	33.2	360	29.3	108	23.3	388	
No	66.8	725	70.7	260	76.7	1275	
New HIV							**<0.001**
Yes	5.6	61	1.6	6	0.4	7	
No	94.4	1024	98.4	362	99.6	1656	
Infectious syphilis							**0.001**
Yes	5.1	55	5.4	20	3.2	54	
No	94.7	1027	94.0	346	95.4	1586	
Not tested	0.3	3	0.5	2	1.4	23	
Infectious hepatitis B							n/a
Yes	1.2	12	0.0	0	0.1	1	
No	59.6	647	53.0	195	40.8	679	
Not tested	39.2	425	47.0	173	59.1	983	
Chlamydia							0.196
Yes	13.8	150	12.5	46	12.4	207	
No	86.2	935	87.5	322	87.6	1456	
Gonorrhea							**0.009**
Yes	16.3	176	16.5	60	12.5	206	
No	93.7	906	83.5	303	87.5	1447	
Diagnosis of any STI within continents							
Eastern Europe	37.0	60/162	36.8	7/19	n/a		0.987
Latin America	36.1	196/543	25.0	8/32	n/a		0.202
Suriname/NL Antilles	31.4	33/105	32.1	34/106	n/a		0.920
Asia	27.7	46/166	19.0	15/79	n/a		0.140
North Africa	23.4	15/64	30.4	34/112	n/a		0.325
Sub-Sahara Africa	22.2	10/45	30.6	11/36	n/a		0.395

*Statistically significant *P* values in bold (*P* < 0.05).

^†^Analysis of variance test to compare means.

^‡^Available from 2018 and onward.

^§^Reported in the last consultation instead of the first consultation.

^¶^Analyzed between August 2019 and onward because of availability of PrEP.

^∥^Missing: 2.3% (25), 1.9% (7), and 1.0% (16), respectively.

Anogenital condom use was reported to be highest among first-generation migrant MSW-MSM (“always” 34.7%) in comparison to the second-generation (22.8%) and Western-born (22.1%) MSW-MSM (*P* < 0.001). First-generation migrants mostly reported having sex with only men (74.5%), whereas a larger proportion of second-generation migrant (40.2%) and Western-born MSW-MSM (42.8%) reported having sex with both men and women (*P* < 0.001). Alcohol/drug use during the past 6 months was reported to be the highest among the Western-born migrant MSW-MSM (50.6% vs. 43.4% and 49.7% for first- and second-generation migrants; *P* = 0.006).

There was a significant difference in diagnosis of any STI (33.2%, 29.3%, 23.3%; *P* < 0.001), new HIV infections (5.6%, 1.6%, 0.4%; *P* < 0.001), infectious syphilis (5.1%, 5.4%, 3.2%; *P* = 0.001), and gonorrhea (16.3%, 16.5%, 12.5%; *P* = 0.009) between the first-generation migrant, second-generation migrant, and Western-born MSW-MSM, respectively. Diagnosis of chlamydia was similar (13.8%, 12.5%, 12.4%; *P* = 0.196) between the 3 groups.

The burden of HIV is high in the first-generation migrants. The 2-yearly newly diagnosed HIV proportions among first-generation migrant MSW-MSM in their first consultation increased from 3.1% (10 of 327) in 2016 to 2017, 5% (14 of 278) in 2018 to 2019, to 7.7% (37 of 480) in 2020 to 2021. Although the first-generation migrants contribute to the majority of the overall HIV burden, a similar increasing trend was seen in the total HIV burden of MSW-MSM over the years (2016–2017: 1.3% [14 of 1078], 2018–2019: 1.8% [16 of 866], 2020–2021: 4% [44 of 1098]).

Between continents, first-generation migrant MSW-MSM from Eastern Europe had the highest burden of any STI (37.0%), followed by MSW-MSM from Latin America (36.1%) and Suriname/NL Antilles (31.4%). Second-generation migrant MSW-MSM from Eastern Europe also had the highest burden of any STI (36.8%), followed by MSW-MSM by Suriname/NL Antilles (32.1%) and Sub-Sahara Africa (30.6%).

The association between diagnosis of any STI in first consultation and migrant group is presented in Table 3. First-generation migrant MSW-MSM had higher odds (adjusted odds ratio, 1.6; 95% confidence interval [CI], 1.3–1.9) of getting an STI diagnosis in the first consultation compared with Western-born MSW-MSM. When excluding HIV from diagnosis with an STI, first-generation migrant MSW-MSM still had higher (adjusted) odds of getting an STI diagnosis in the first consultation compared with Western-born MSW-MSM.

**TABLE 3:** Association Between Diagnosis of Any Sexually Transmitted Infections (STI) in First Consultation and Migrant Groups Who Visited an STI Clinic From 2016 to 2021 in the Netherlands

Main Determinant	OR (95% CI)	aOR* (95% CI)	Any STI Excluding HIV, aOR* (95% CI)
First-generation migrant MSW-MSM	1.6 (1.4–1.9)^†^	1.6 (1.3–1.9)^†^	1.4 (1.2–1.7)^†^
Second-generation migrant MSW-MSM	1.4 (1.16–1.8)^‡^	1.2 (0.95–1.6)	1.2 (0.9–1.5)
Western-born MSW-MSM	1 (ref)	1 (ref)	1 (ref)

*Adjusted for age and urbanity of STI clinic region.

^‡^*P* < 0.01.

^†^*P* < 0.001.

Incidence of a first repeat consultation was 25.8 per 100 person-years (95% CI, 23.5–28.2) among first-generation migrant MSW-MSM, 19.7 per 100 person-years (95% CI, 16.6–23.1) among second-generation migrant MSW-MSM, and 16.7 (95% CI, 15.4–18.1) among Western-born MSW-MSM.

**Figure 1:**
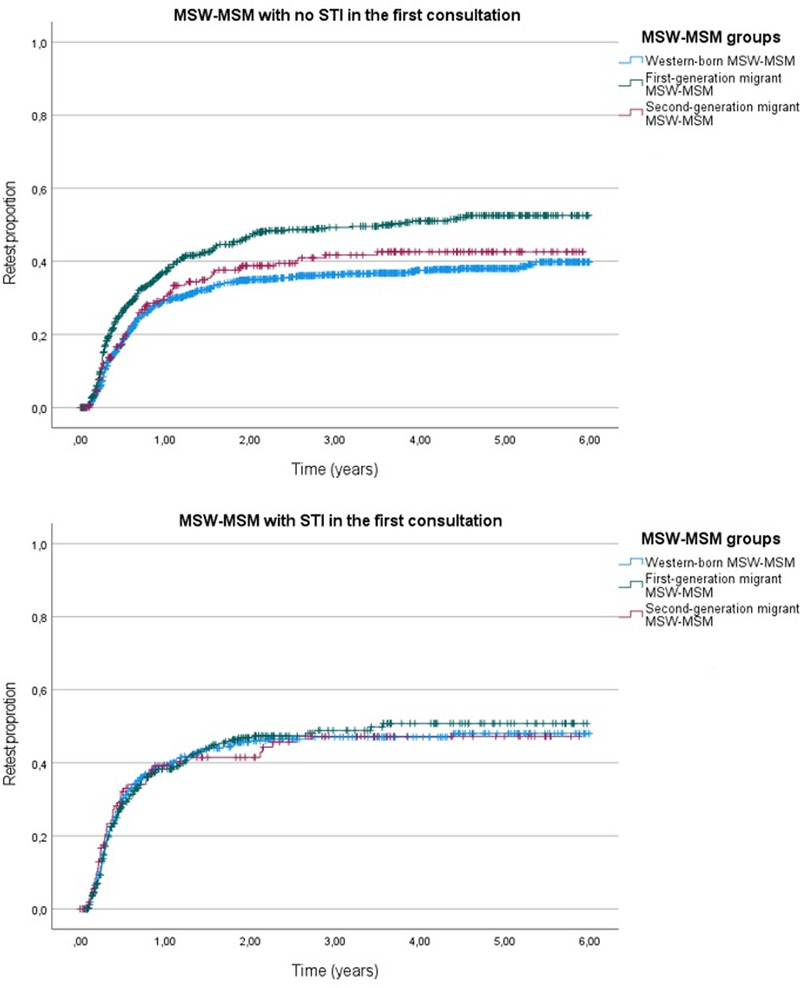
Survival curve of the proportion of repeat consultations in the years 2016 to 2021 among first-, second-generation, and Western-born MSW-MSM (n = 3116), stratified by STI in the first consultation.

A survival curve of repeat consultations among the 3 MSW-MSM groups over the years 2016 to 2021, stratified for no STI in the first consultation (*P* < 0.001) and STI in the first consultation (*P* = 0.983), is presented in Figure 1. First-generation migrant MSW-MSM (in reference to Western-born) have an adjusted HR of 1.5 (95%CI, 1.3–1.8; *P* < 0.001) of having a repeat consultation at any time, when stratified for not having an STI in the first consultation.

Sexually transmitted infection/HIV burden for one-time testers and repeat testers among first-, second-generation and Western-born MSW-MSM is presented in Supplementary Table 1, http://links.lww.com/OLQ/B14. Among first-generation migrant MSW-MSM, 57.4% (623) did not return for repeat testing. Of these one-time testers, 33.7% (210) were diagnosed with an STI. Sixty-three percent (232) of second-generation migrant MSW-MSM did not return for repeat testing, of which 28.4% (66) of the one-time testers were diagnosed with an STI. Of the Western-born MSW-MSM, 1072 (64.5%) did not return for repeat testing, of which 20.8% (223) were diagnosed with an STI. Among both first-generation and second-generation migrant MSW-MSM, new HIV infections were more prevalent in one-time testers, compared with the first consultation and the first repeat consultation of repeat testers. A similar pattern was identified for infectious hepatitis B in first-generation MSW-MSM. Overall, burden of STI decreased in the first repeat consultation compared with the first consultation for the repeat testers among first- and second-generation MSW-MSM, but not among Western-born MSW-MSM.

## DISCUSSION

In this large study, we assessed sociodemographic characteristics of MSW-MSM, burden and chance of STI/HIV, and sexual health care–seeking behavior of first- and second-generation migrant and Western-born MSW-MSM in the Netherlands.

During a 6-year study period, 0.9% of all STI clinic consultations in the Netherlands were of (migrant) MSW-MSM patients. Even though this is a small proportion of all STI clinic consultations, the burden of STI was high among all 3 MSW-MSM groups, but highest among first- and second-generation migrant MSW-MSM. In addition, first-generation migrant MSW-MSM had a substantially higher chance of an STI diagnosis compared with Western-born MSW-MSM.

The burden of new HIV infections, infectious syphilis, and gonorrhea is higher among first- and second-generation migrant MSW-MSM compared with Western-born MSW-MSM, although the chlamydia burden is similar among all 3 MSW-MSM groups. Both first- and second-generation migrants from Eastern Europe have the highest burden of STI. A Dutch study compared the burden of STI among FSW, MSM, and MSW, concluding that MSW tested positive for an STI in 40% of consultations, which was significantly higher than in FSW (4%) and MSM (14%).^14^ Furthermore, the male sex workers included in that study were predominantly Eastern European migrants, which is in line with our study finding that the highest burden of STI is found among Eastern European migrant MSW-MSM.^14^ In this study, alcohol/drug use in the past 6 months was reported by 43.4% to 50.6% of MSW-MSM, which is similar to a large European study and a Dutch study reporting 39.4% to 40.5% for drug and 37.9% to 56.5% for alcohol use among MSW-MSM.^15,16^ These studies also reported condomless anal sex by 19% to 64.3% of MSW-MSM in the past 6 to 12 months, in comparison to 64.1% to 75.1% in this study reporting “never” and “not always” using a condom during anogenital sex in the past 6 months.^15,16^

First-generation migrant MSW-MSM seemed to be more likely to have a repeat STI consultation compared with Western-born MSW-MSM when they had no STI diagnosis in the first consultation. This indicates that once first-generation migrant MSW-MSM have visited the STI clinic, they are not more lost to care than other MSW-MSM groups. In a study from the United Kingdom, health care–seeking behavior did not vary between migrant and UK-born male sex workers,^17^ whereas we established that there is a difference in health care–seeking behavior, that is, repeat consultations. There was no difference in health care–seeking behavior between the 3 MSW-MSM groups when diagnosed with an STI in the first consultation.

First-generation migrant MSW-MSM had a higher burden of any STI and HIV in their only/first consultation compared with the repeat consultation. A similar pattern was found in a Spanish study among male sex workers, of which 67% was migrant.^18^ It could be hypothesized that this reflects a higher STI prevalence from their country of origin.^19^ However, another systematic review suggested to have found no evidence of migrant sex workers bringing STI to destination countries.^20^ In addition, among first- and second-generation MSW-MSM, STI diagnoses decreased in the repeat consultation compared with the first consultation for the repeat testers, indicating a possible effect of the STI clinic's counseling and highlighting the relevance of low-threshold STI testing and treatment for migrant MSW-MSM. Furthermore, it remains important to also focus on Western-born MSW-MSM and provide tailored risk-reduction strategies in counseling to as well decrease the STI burden in this group.

### Strengths and Limitations

To our knowledge, this is the first study including MSW-MSM distinguishing first- and second-generation migrants and making a comparison between migrant generations and Western-born with regard to STI burden and health care–seeking behavior. Furthermore, because of the long study period of 6 years, we were able to include a large number of (migrant) MSW-MSM nationally.

In this study, we use electronic patient registry's coded surveillance consultations from 2016 to 2021, which has limitations. First, because of the chosen inclusion period, what is considered a first STI consultation of an MSW-MSM could possibly be a repeat consultation. Second, we might overestimate the number of unique patients because a patient will be registered as a new patient in a different (STI clinic) region and could also have multiple files per clinic because of anonymous registration being allowed. Third, missing data hampered some analyses, as we were unable to adjust for certain sociodemographic and behavioral characteristics. Fourth, we do not have data on STI consultations at other health care providers, such as general practitioners, limiting our study's generalizability. However, because general practitioners predominantly perform STI tests for low-incidence groups, this limitation might be small.^21,22^ Fifth, because STI such as HIV and syphilis can remain undetected for extended periods, the period during which STIs were acquired may be much longer for the first consultation compared with the repeat consultation. This time discrepancy between infection acquisition and diagnosis is a limitation in our approach of comparing first and repeat consultations and should be understood when interpreting our study findings. Last, the electronic patient registry did not facilitate proper registration of transgender persons. Consequently, we were not able to distinguish between MSM and transgender sex workers in our data. Therefore, an unknown part of our study population identifies as transgender. Although not representative for the whole country, data from the Prostitution and Health Center in Amsterdam show that 6.4% of all visiting sex workers were MSW-MSM and 2.6% were transgender persons.^16^ We expect these percentages to represent the upper range proportion of transgender sex workers. Furthermore, MSW-MSM had a higher STI positivity (29.0%) in comparison to transgender sex worker (26.7%), and both groups had a similar proportion of people with HIV (20.3% vs. 20.0%),^16^ suggesting that the impact on our results of this limitation is small.

### Implications for Care

In this study, we established that new HIV diagnoses among first-generation MSW-MSM have increased from 3.1% in 2016 to 2017 to 7.7% in 2020 to 2021 as well as in the total population of MSW-MSM. In contrast, among the general MSM population, the trend has been decreasing.^23^ Because the number of HIV-tested MSW-MSM was similar over the years, the rising HIV diagnoses seem to not be a matter of increased reach of HIV testing but rather a genuine rise in HIV prevalence among the MSW-MSM population. Although it is promising that preexposure prophylaxis (PrEP) uptake in this study was equal among the MSW-MSM groups and that migrant MSW-MSM seemingly have similar access to PrEP care to Western-born MSW-MSM, it remains concerning that in these high-incidence populations of sex workers with a rising HIV-prevalence, PrEP uptake is lower than that of non–sex worker MSM.^24^

Furthermore, the first-generation migrants mostly had sex with only men (74.5%), whereas 40.2% and 42.8% of second-generation migrant and Western-born MSW-MSM had sex with both men and women. Consequently, second-generation migrant and Western-born MSW-MSM could be a bridge population of STI transmission to female sex partners, as previously noted in another Dutch MSW study.^14^

In this study, we used repeat consultations as a measure for health care–seeking behavior, because general data on the total number of (migrant) MSW-MSM in the Netherlands are absent, which hinders the capability to calculate consultation rates for each MSW-MSM group.

Research suggests that MSW-MSM are less visible than FSWs, their numbers are smaller, and the group is understudied.^5,25^ Access of MSW-MSM to (sexual) health care is hampered by multilayered stigma: stigma related to sexuality, gender identity, HIV status, sex worker status, and internalized stigma.^26^ Other barriers to access of care are confidentiality concerns, a lack of self-identification as homosexual and sex worker, sexual health literacy, fatalism, and structural barriers.^26,27^ Trust between MSW-MSM and the sexual health care provider has been identified as an important facilitator.^27^

An even greater challenge for sexual health care providers is reaching migrant MSW-MSM. Reasons for hampered health care–seeking behavior in migrant groups are multifold: fear and distrust of health care institutions, as well as social and cultural discrimination, language barriers, provider bias (or the perception that these may exist), and affordability (e.g., travel cost to the clinic, costs of health care itself).^28,29^

Unfortunately, the (public) STI clinics in the Netherlands do not have an optimal strategy, or the funding or capacity to adequately reach migrant MSW-MSM. Most STI clinics perform Internet fieldwork on websites where sex workers advertise and offer low threshold STI testing, but this might not be enough to bridge the testing gap. Other ways to reach migrant MSW-MSM is by providing testing distributed by lay providers or social network testing.^30,31^ These methods lower the testing threshold by being free-of-charge, the absence of a waiting list or triaging when booking an appointment, improving privacy, not requiring disclosure of sexual identity, and less experienced stigma related to sexual practices and sex work.^30,31^ Although these initiatives are a valuable addition to the low-threshold care provided by the STI clinics, they are not yet common practice in the Netherlands.

### Conclusions

The high STI/HIV burden among all 3 MSW-MSM groups and substantially higher odds of STI for first-generation migrant compared with Western-born MSW-MSM highlight the relevance of low-threshold STI testing and treatment for (migrant) MSW-MSM. Health care–seeking behavior is higher among first-generation migrant MSW-MSM compared with Western-born MSW-MSM, and thus, they are not more lost to care. Future studies are needed to close the knowledge gap on (migrant) MSW-MSM and develop tailored risk-reduction strategies, including, for example, promoting PrEP use, to lower the substantial STI/HIV burden among these MSW-MSM groups.
